# Supplementary material for: Meta-Analysis and Gene Set Enrichment Relative to ER Status Reveal Elevated Activity of MYC and E2F in the “Basal” Breast Cancer Subgroup
Source: PLoS One. 2009 Mar 9;4(3):e4710. doi: 10.1371/journal.pone.0004710 (PMC2650420; doi:10.1371/journal.pone.0004710)
Supplement: Dataset S1 — GSEA results from first screen. The file contains a zipped archive of the HTML results of the second GSEA screen. Reports for enriched or depleted datasets may be accessed from the “index.html” link within the my_analysis.GseaPreranked.1219975950625.rpt.zip ZIP archive. Enrichment plots are available for each of the 50 most enriched and depleted gene sets. (13.32 MB ZIP) [file pone.0004710.s001.zip › my_analysis.GseaPreranked.1219975950625.rpt.zip!//ET743_RESIST_UP.html]

Details for gene set ET743\_RESIST\_UP[GSEA]

|  || Dataset | zSco.ERneg.to.ERpos\_collapsed |
| Phenotype | NoPhenotypeAvailable |
| Upregulated in class | na\_neg |
| GeneSet | ET743\_RESIST\_UP |
| Enrichment Score (ES) | -0.5456215 |
| Normalized Enrichment Score (NES) | -1.6816726 |
| Nominal p-value | 0.02710027 |
| FDR q-value | 0.07430907 |
| FWER p-Value | 0.985 |
Table: GSEA Results Summary

  

Fig 1: Enrichment plot: ET743\_RESIST\_UP      
 Profile of the Running ES Score & Positions of GeneSet Members on the Rank Ordered List

  

| PROBE | GENE SYMBOL | GENE\_TITLE | RANK IN GENE LIST | RANK METRIC SCORE | RUNNING ES | CORE ENRICHMENT || 1 | YWHAQ |  |  | 774 | 3.803 | 0.0500 | No |
| 2 | TJP2 |  |  | 3237 | 1.822 | -0.0832 | No |
| 3 | VBP1 |  |  | 3382 | 1.758 | -0.0440 | No |
| 4 | CSF2RA |  |  | 3824 | 1.581 | -0.0322 | No |
| 5 | DNAJB1 |  |  | 5509 | 0.971 | -0.1312 | No |
| 6 | DUT |  |  | 6839 | 0.539 | -0.2157 | No |
| 7 | IFI6 |  |  | 7158 | 0.433 | -0.2273 | No |
| 8 | IFIT1 |  |  | 7884 | 0.152 | -0.2775 | No |
| 9 | RAD51C |  |  | 8329 | -0.007 | -0.3107 | No |
| 10 | AGT |  |  | 10424 | -1.102 | -0.4367 | No |
| 11 | GLUL |  |  | 11874 | -2.404 | -0.4772 | Yes |
| 12 | ZNF24 |  |  | 12005 | -2.565 | -0.4140 | Yes |
| 13 | GDI1 |  |  | 12108 | -2.714 | -0.3445 | Yes |
| 14 | ARHGDIB |  |  | 12440 | -3.165 | -0.2793 | Yes |
| 15 | ATP6V0C |  |  | 12506 | -3.271 | -0.1912 | Yes |
| 16 | AKAP12 |  |  | 12700 | -3.622 | -0.1026 | Yes |
| 17 | CRIP2 |  |  | 13180 | -5.244 | 0.0105 | Yes |
Table: GSEA details [plain text format]

  

Fig 2: ET743\_RESIST\_UP: Random ES distribution      
 Gene set null distribution of ES for **ET743\_RESIST\_UP**
